# Clinical and microbiological characteristics of persistent *Staphylococcus aureus* bacteremia, risk factors for mortality, and the role of CD4^+^ T cells

**DOI:** 10.1038/s41598-024-66520-0

**Published:** 2024-07-05

**Authors:** Eunmi Yang, Yeong Geon Cho, Eunsil Kim, Euijin Chang, Seongman Bae, Jiwon Jung, Min Jae Kim, Yong Pil Chong, Sung-Han Kim, Sang-Ho Choi, Sang-Oh Lee, Yun Shin Chung, Yang Soo Kim

**Affiliations:** 1 Division of Infectious Diseases, Seoul Medical Center, Seoul, South Korea; 2 Center for Antimicrobial Resistance and Microbial Genetics, University of Ulsan College of Medicine, Seoul, South Korea; 3 Asan Institute for Life Science, Asan Medical Center, Seoul, South Korea; 4 Division of Infectious Diseases, Asan Medical Center, University of Ulsan College of Medicine, 88, Olympic-ro43-gil, Songpa-gu, Seoul, 05505 South Korea

**Keywords:** Immunology, Microbiology, Medical research

## Abstract

This study evaluated the determinants of mortality and the T cell immune response in patients with persistent *Staphylococcus aureus* bacteremia (SAB). This was a prospective cohort study and patients with confirmed SAB were enrolled from 2008 to 2020. We compared clinical, microbiological, and genotypic features between surviving and deceased patients with persistent SAB. The concentrations of cytokines and the proportions of IFN-γ secreting CD4^+^ T cells were measured serially during the bacteremia period. Of the 1760 patients, 242 had persistent bacteremia (PB), and 49 PB patients died within 30 days. In the multivariate analysis, the APACHE II score and female sex were independently associated with 30 days mortality. The level of IL-10 was significantly increased in the plasma of patients with a high Pitt bacteremia score and those who died within 12 weeks from the index day. The proportion of IFN-γ-secreting CD4^+^ T cells were the highest just before the positive-to-negative conversion of blood cultures in patients with a low Pitt bacteremia score and those who survived for 12 weeks. The level of IL-10 is correlated with clinical outcomes in PB patients. IFN-γ secreting CD4^+^ T cells might play a pivotal role in SAB PB.

## Introduction

*Staphylococcus aureus* is a leading cause of serious bacterial infections^1,2^. *S. aureus* bacteremia (SAB) may persist in some patients despite appropriate antibiotic therapy. The use of intravascular catheters or prosthetic devices, metastatic infection, methicillin resistance, vancomycin minimal inhibitory concentration (MIC), and accessory gene regulator (*agr)* dysfunction have been suggested as risk factors for persistent bacteremia (PB)^3–6^. Several reports have identified clinical outcomes associated with PB compared with resolving bacteremia (RB). In patients with SAB, PB is typically associated with complications of bacteremia, longer hospitalization, and increased mortality than RB^4,6–9^. Factors that predict mortality must be elucidated to determine the optimal strategy for treating *S. aureus* PB.

The outcome of *S. aureus* infection could be influenced by the host immune response to *S. aureus*. The cytokine response to *S. aureus* infection appears to be related to clinical outcomes. IL-6 is an early inflammatory marker of complicated SAB, and IL-10 is associated with mortality and PB^10–13^. Also, previous studies have attempted to establish the contribution of T cell immunity during *S. aureus* infection, along with efforts to develop a vaccine against *S. aureus* infection^14,15^. CD4^+^ T helper (Th) cell immunity might be important for fighting *S. aureus* infections in humans^16^. In particular, Th1 cells produce IFN-γ, which promotes bacterial elimination inside macrophage phagosomes and plays an important role in immunological and inflammatory processes during *S. aureus* infection^14,16–18^.

Although there have been several reports evaluating the mortality of SAB and risk factors for PB, the factors affecting the mortality of patients with PB remain unclear. In addition, most studies of T cell immunity during *S. aureus* infection have been performed in murine models, not in humans^17–19^. The goal of this study was to evaluate the clinical, microbiologic, and genotypic risk factors of 30 days mortality in patients with *S. aureus* PB in a prospective cohort over a 13 years period, and to investigate the cytokine kinetics in the plasma and the T cell immune response in a clinical setting of *S. aureus* PB.

## Patients and methods

### Human studies

Plasma and peripheral blood mononuclear cells (PBMCs) were obtained from patients at the Asan Medical Center as part of the *S. aureus* bacteremia and immune response studies. This study was conducted at the Asan Medical Center, a 2700-bed tertiary-care referral center in South Korea, from July 2008 to December 2020. All adult patients with SAB were prospectively enrolled in a cohort and observed over 12 weeks. Patients were excluded from the analysis if they had polymicrobial bacteremia, had been discharged before obtaining positive blood culture results, or had SAB within the previous three months. Demographic characteristics, underlying diseases or conditions, the severity of underlying diseases, the severity of bacteremia, place of infection, site of infection, presence of a central venous catheter (CVC) or prosthetic device, patient management, and clinical outcomes were recorded. Acute Physiology and Chronic Health Evaluation II (APACH II) and Pitt bacteremia scores were calculated on the day of the first positive blood culture to assess the severity of bacteremia^20,21^. The Charlson comorbidity index was employed to determine the severity of comorbid conditions^22^.

### Study definitions

PB was defined as bacteremia for ≥ 7 days while receiving appropriate antibiotics therapy. The place of acquisition was categorized as community-acquired, healthcare-associated, or hospital-acquired infection^23^. Infective endocarditis was defined according to the modified Duke criteria^24^. Two sets of cultures were obtained to diagnose incident bloodstream infections. The index day was defined as the day on which the first positive blood culture was obtained. Empirical antibiotic treatment was considered appropriate if at least one antibiotic effective against the SAB isolate was started within 24 h after the index blood culture. Prosthetic devices included orthopedic devices, cardiovascular electronic devices, prosthetic valves, and vascular grafts. Septic shock was defined as sepsis with persisting hypotension that required vasopressors to maintain the patient’s blood pressure despite adequate fluid resuscitation^25^. The concentrations of cytokines and the proportion of IFN-γ secreting CD4 + T cells were compared according to the patient's bacteremia severity scores and 12-week mortality. The patients were divided into groups: Group 1 had Pitt bacteremia score < 4, and Group 2 had Pitt bacteremia score ≥ 4. Group 3 was patients who survived for 12 weeks from the index day, and Group 4 was patients who died within 12 weeks.

### Microbiological analysis

All *S. aureus* isolates were identified by standard methods. The first blood isolate obtained from the patient was used for the microbiological and molecular assessments. The minimum inhibitory concentration (MIC) of vancomycin was determined using a broth microdilution method (BMD) according to the standard protocol. Methicillin resistance was confirmed by detecting the *mec*A gene via polymerase chain reaction (PCR).

The staphylococcal cassette chromosome *mec* (SCC*mec*) type and *agr* genotype were identified as previously described^26,27^. The *agr* function was determined by the δ-hemolysin activity as described elsewhere^28^. Multi-locus sequence typing (MLST) was performed for all strains as described previously^29^. MLST allele names and STs were derived from the MLST database (http://www.mlst.net). Clonal complexes (CCs) were assigned to groups of isolates that shared six of seven alleles via eBURST (http://eburst.mlst.net).

### Cytokine assays

The concentrations of IL-6, IL-10, IL-17A, IL-12p70, IL-13, IL-4, TNF-α, and IFN-γ in plasma were measured with a ProcartapPlex Multiplex Immunoassay (Thermo Fisher Scientific, Waltham, MA, USA) according to the manufacturer’s instructions. If the level for a particular cytokine was below the detectable limit, the concentration was recorded as 0.

### Peripheral blood mononuclear cell isolation

Venous blood was collected in heparinized vacutainer tubes (BD Biosciences Pharmingen, San Jose, CA, USA) and diluted with 1 × PBS (HyClone Laboratories, Inc., South Logan, UT, USA). The PBMCs were isolated by Ficoll-Hypaque (GE Healthcare Bio-Sciences AB, Uppsala, Sweden) gradient separation. The PBMCs were used directly or washed in RPMI-1640 and diluted in freezing medium containing 40% RPMI-1640, 50% fetal calf serum (Gibco by Invitrogen, Carlsbad, CA, USA), and 10% dimethyl sulfoxide (Sigma Aldrich, St. Louis, MO, USA), gradually frozen in a freezing container (Mr. Frosty, Nalgene Cryo 1 °C; Nalgene Co., Rochester, NY, USA), and stored in liquid nitrogen until analyzed.

### In vitro activation, intracellular staining, and flow cytometry analysis of PBMCs

PBMCs were thawed and washed before counting and the exclusion of non-viable cells by trypan blue staining. Cells were resuspended to a final density of 5 × 10^5^–1 × 10^6^/well and stimulated with 5 × 10^7^–1 × 10^8^ CFU/mL of heat-killed *S. aureus* (the multiplicity of infection was 1:100) for 4 days. The cell viability was confirmed by Near-IR fluorescent reactive dye (Invitrogen), and then the cells were stained for CD3 (BD horizon, clone UCHT1), CD4 (eBioscience, clone RPA-T4), and CD8 (eBioscience, clone RPA-T8). The cells were fixed and permeabilized using the BD Cytofix/Cytoperm Fixation and Permeabilization Kit, followed by intracellular staining with fluorochrome-conjugated antibodies against IFN-γ (eBioscience, clone4S.B3). Flow cytometric data were acquired with a BD FACSCanto II and analyzed using FlowJo software version 10.8.1 (Becton, Dickinson & Company; www.flowjo.com)^30^.

### Statistical analysis

We compared surviving and deceased patients with PB. Categorical variables were compared using the Chi-square test or Fisher’s exact test, as appropriate. Continuous variables were compared using Student’s *t* test and the Mann–Whitney U-test. Logistic regression was used to assess risk factors associated with PB mortality. Risk factors for 30 days mortality from PB were assessed using univariate analysis, and statistically significant variables were included in the multivariate analysis. Two-way ANOVA with Bonferroni post-tests was used to analyze the cytokine kinetics. A two-tailed *p* value < 0.05 was considered significant. All statistical analyses were performed using IBM SPSS Statistics for Windows, version 25 (IBM Corp., Armonk, NY, USA) and GraphPad Prism version 9.4.1 (GraphPad Software, Inc., San Diego, CA, USA; www.graphpad.com).

### Ethics declarations

The study was approved by the Institutional Review Board (IRB) of Asan Medical Center (protocol No. 2013-1002). Informed consent was obtained from all individual participants included in the research. The study was performed in accordance with the Helsinki Declaration.

## Results

### Clinical characteristics and outcomes of patients with PB

From July 2008 to December 2020, 1760 episodes of SAB were reported. Among these 1760 episodes, 242 (13.8%) developed PB with a median duration of 11 days (interquartile range [IQR], 8–16 days). The following types of PB infections were identified: CVC-related infections (33.5% [81/242]), bone and joint infections (20.2% [49/242]), endocarditis (9.9% [24/242]), skin and soft tissue infections (7.0% [17/242]), pneumonia (2.1% [5/242]), and primary bacteremia (7.0% [17/242]). The rate of metastatic infections was 48.3% [117/242].

Comparisons of the demographic and clinical characteristics of the deceased and surviving patients are shown in Table 1. A total of 49 (20.2%) patients died within 30 days after the index day, and 193 (79.8%) patients survived. The deceased patients were more likely to have liver cirrhosis (30.6% [15/49] vs. 11.9% [23/193], *p* < 0.01), a high Charlson comorbidity score (median score 4 vs. 3,* p* = 0.03), and a high APACHE II score (median score 19 vs. 16,* p* = 0.01) than the surviving patients. The surviving patients, on the other hand, were more commonly male (69.9% [135/193] vs. 51.0% [25/49], *p* = 0.01), had a community-acquired infection (21.2% [41/193] vs. 4.1% [2/49], *p* = 0.01), diabetes mellitus (39.4% [76/193] vs. 20.4% [10/49], *p* = 0.01), metastatic bone and joint infections (15.0% [29/193] vs. 4.1% [2/49], *p* = 0.04), and it took longer to remove an eradicable focus (2 days vs. 1 day,* p* = 0.04).

**Table 1:** Demographic and clinical characteristics of patients with persistent *S. aureus* bacteremia.

Characteristic	Deceased patients (n = 49) no. (%)	Surviving patients (n = 193) no. (%)	Odds ratio (95% CI)	*p* value
Age (yr), median (IQR)	61 (52.5–75.0)	65 (55.5–71.0)	1.00 (0.96–1.05)	0.51
Male	25 (51.0)	135 (69.9)	0.46 (0.24–0.86)	0.01
Place of acquisition				
Community-onset	20 (40.8)	104 (53.9)	0.59 (0.31–1.10)	0.10
Community-acquired	2 (4.1)	41 (21.2)	0.15 (0.04–0.66)	0.01
Healthcare-associated	18 (36.7)	63 (32.6)	1.21 (0.63–2.32)	0.59
Hospital-acquired	29 (59.2)	89 (46.1)	1.71 (0.91–3.23)	0.10
Underlying disease				
Diabetes mellitus	10 (20.4)	76 (39.4)	0.39 (0.18–0.83)	0.01
Solid tumor	18 (36.7)	63 (32.6)	1.21 (0.63–2.32)	0.59
Liver cirrhosis	15 (30.6)	23 (11.9)	3.28 (1.55–6.93)	< 0.01
End-stage renal disease	6 (12.2)	29 (15.0)	0.79 (0.31–2.03)	0.62
Hypertension	19 (38.8)	93 (48.2)	0.69 (0.36–1.30)	0.24
Cardiovascular disease	7 (14.3)	20 (10.4)	1.45 (0.58–3.65)	0.44
Hematologic malignancy	2 (4.1)	11 (5.7)	0.71 (0.15–3.30)	> 0.99
Prior antibiotic use within 1 mo	26 (53.1)	84 (43.5)	1.48 (0.79–2.78)	0.23
Methicillin-resistant isolate	36 (73.5)	149 (77.2)	0.84 (0.41–1.71)	0.58
Charlson comorbidity index, median (IQR)	4 (2–6)	3 (1–4)	1.12 (0.94–1.34)	0.03
APACHE II, median (IQR)	19 (14.5–24.5)	16 (11.5–60.0)	1.05 (0.97–1.15)	0.01
Pitt bacteremia score, median (IQR)	1 (0–3.5)	1 (0–2)	1.15 (0.86–1.52)	0.19
Septic shock	10 (20.4)	20 (10.4)	2.23 (0.97–5.14)	0.06
C-reactive protein (mg/dL)(IQR)	13.6 (7.4–23.0)	13.9 (7.2–23.2)	1.02 (0.97–1.06)	0.92
*agr* dysfunction	28 (57.1)	106 (54.9)	1.11 (0.59–2.08)	0.78
Central venous catheter	19 (38.8)	75 (38.9)	1.00 (0.53–1.91)	0.99
Prosthetic device^a^	11 (22.4)	45 (23.3)	0.93 (0.44–1.97)	0.90
Characteristics of infection				
CVC-related infection	16 (32.7)	65 (33.7)	0.96 (0.49–1.88)	0.89
Removal of CVC	15/16 (93.8)	65/65 (100)	NA	0.25
Bone and joint infection	6 (12.2)	43 (22.3)	0.49 (1.20–1.23)	0.12
Primary bacteremia	5 (10.2)	12 (6.2)	1.58 (0.54–4.67)	0.35
Skin and soft tissue	3 (6.1)	14 (7.3)	0.84 (0.23–3.04)	> 0.99
Infective endocarditis^b^	6 (12.2)	18 (9.3)	1.36 (0.51–3.64)	0.59
Surgical site infection	2 (4.1)	10 (5.2)	0.78 (1.67–3.70)	> 0.99
Pneumonia	3 (6.1)	2 (1.0)	6.26 (1.02–38.56)	0.06
Peripheral catheter-related infection	3 (6.1)	4 (2.1)	3.10 (0.67–14.32)	0.15
Metastatic infection	18 (36.7)	99 (51.3)	0.56 (0.29–1.06)	0.07
Lung (septic pneumonia)	5 (10.2)	30 (15.5)	0.62 (0.23–1.70)	0.34
Skin and soft tissue	4 (8.2)	37 (19.2)	0.38 (0.13–1.12)	0.07
Bone and joint	2 (4.1)	29 (15.0)	0.24 (0.06–1.05)	0.04
Central nervous system	6 (12.2)	14 (7.3)	1.79 (0.65–4.94)	0.25
Eye (endophthalmitis)	4 (8.2)	14 (7.3)	1.14 (0.36–3.64)	0.77
Cardiac valve (endocarditis)	4 (8.2)	10 (5.2)	1.64 (0.49–5.46)	0.49
Eradicable focus	26 (53.1)	122 (63.2)		0.19
Removal of eradicable focus^c^	24/26 (92.3)	110/122 (90.2)	1.31 (0.28–6.23)	> 0.99
Time of removal (d), median (IQR)	1 (0–3)	2 (0–8)	1	0.04

IQR, interquartile range; APACHE II, acute physiology and chronic health evaluation II; CVC, central venous catheter.

^a^Includes orthopedic devices (12 patients), cardiovascular implantable electronic devices (3 patients), prosthetic valves (12 patients), and vascular grafts (27 patients).

^b^Echocardiography was performed in 94.6% (229/242) of patients with persistent bacteremia.

^c^Eradicable focus refers to a removable source of infection, such as a vascular catheter, vascular graft, prosthetic device, or abscess.

### Risk factors for 30 days mortality of patients with PB

A multivariate logistic regression analysis was performed to identify independent risk factors associated with 30 days mortality from PB (Table 2). The multivariate analysis indicated that the APACHE II score (adjusted odds ratio [aOR], 1.10; 95% confidence interval [CI] 1.03–1.17) and female sex (aOR 3.84; 95% CI 1.40–10.54) were independent risk factors for 30 days mortality from PB.

**Table 2:** Multivariate analysis of the risk factors for 30 days mortality in patients with persistent *S. aureus* bacteremia.

Characteristic	Univariate analysis			Multivariate analysis^a^	
	Deceased patients (n = 49)	Surviving patients (n = 193)	*p* value	Odds ratio (95% CI)	*p* value
Age	61 (52.5–75.0)	65 (55.5–71.0)	0.51		
APACHE II	19 (14.5–24.5)	16 (11.5–20.0)	0.05	1.10 (1.03–1.17)	0.01
Community-acquired infection	2 (4.1)	41 (21.2)	0.01		
Diabetes mellitus	10 (20.4)	76 (39.4)	0.01		
Liver cirrhosis	15 (30.6)	23 (11.9)	< 0.01		
Male	25 (51.0)	135 (69.9)	0.05	0.26 (0.10–0.72)	0.01
Metastatic infection	18 (36.7)	99 (51.3)	0.07		
Primary bacteremia	5 (10.2)	12 (6.2)	0.35		
Methicillin-resistant isolate	36 (73.5)	149 (77.2)	0.58		
Had a CVC or prosthetic device	24 (49.0)	105 (54.4)	0.30		
Time of removal eradicable focus	1 (0–3)	2 (0–8)	0.04		

^a^This model fits the data well in terms of discrimination (C-statistic 0.769) and calibration (Hosmer–Lemeshow goodness-of-fit statistic 9.492; *p* = 0.303).

### Microbiological characteristics and genotypic studies in MRSA PB

The microbiological characteristics and genotypic studies of the methicillin-resistant *Staphylococcus aureus* (MRSA) PB are shown in Supplementary Tables S1 and S2, respectively. Of the 242 episodes of PB, 185 (76.4%) were MRSA. Of the 185 patients with MRSA bacteremia, 36 (19.5%) died within 30 days from the index day, and 149 (80.5%) survived. A total of 145 (78.4%) patients were initially treated with vancomycin. Most MRSA isolates (71.4%) had vancomycin MIC of ≤ 1.0 mg/L. Surviving patients were more likely to have a vancomycin MIC of ≤ 1.0 mg/L than deceased patients (*p* = 0.02). Common genotypes of MRSA included ST5-SCC*mec* II (58.9% [109/185]) and ST72-SCC*mec*IV (28.6% [53/185]), and common *agr* genotypes were group I (37.8% [70/185]) and group II (58.4% [108/185]). A total of 120 isolates (64.9%) showed *agr* dysfunction. There were no significant differences in MLST type, SCC*mec* type, *agr* genotype, or *agr* dysfunction between the deceased and surviving groups.

### Risk factors for 30 days mortality from MRSA PB

A multivariate logistic regression analysis was employed to identify independent risk factors associated with 30 days mortality from MRSA PB (Supplementary Table S3). The multivariate analysis indicated that the APACHE II score (aOR 1.07; 95% CI 1.01–1.13), liver cirrhosis (aOR 3.77; 95% CI 1.43–9.95), and a vancomycin MIC of ≥ 1.5 mg/L (aOR 3.17; 95% CI 1.39–7.25) were independent risk factors for 30 days mortality in MRSA PB.

### Kinetics of IL-6 and IL-10 in patients with PB

**Figure 1:**
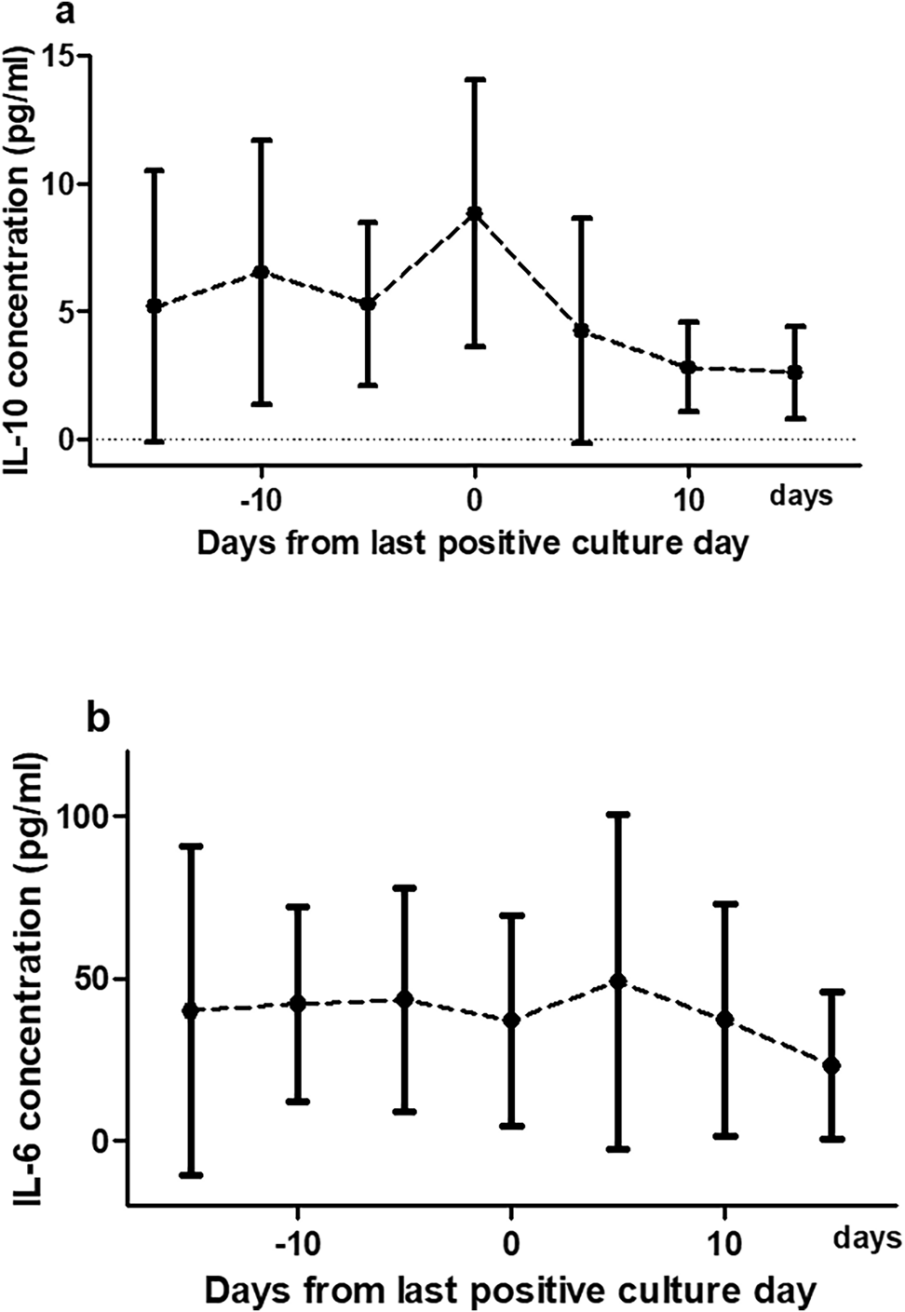
Cytokine kinetics during the bacteremia period. Plasma concentrations of IL-10 and IL-6 were analyzed in serial blood samples from 19 and 22 patients, respectively. (**a**) and (**b**) are the mean concentrations of IL-10 and IL-6 during the bacteremia period. Day 0 is the last positive culture day.

Among 242 patients with PB, 22 patients had blood samples collected periodically. Clinical backgrounds and outcomes of the 22 patients are listed in Supplementary Table S4.Their IL-6, IL-10, IL-17A, IL-12p70, IL-13, IL-4, and TNF-α plasma concentrations were analyzed. One patient died within 30 days, and three patients died within 12 weeks of the index day. IL-12p70, IL-13, and IL-4 levels were undetectable in most patients. IL-17A and TNF-α levels were measured in only eight and two patients, respectively. Therefore, IL-17A, IL-12p70, IL-13, IL-4, and TNF-α were excluded from the analysis. The IL-10 level could not be detected in three patients, and thus only 19 patient samples were available for the analysis of IL-10. Changes in the plasma levels of IL-10 and IL-6 during bacteremia are presented in Fig. 1. The cytokine kinetics were analyzed according to the last positive culture day. Plasma concentrations of IL-10 and IL-6 generally decreased during the study.

**Figure 2:**
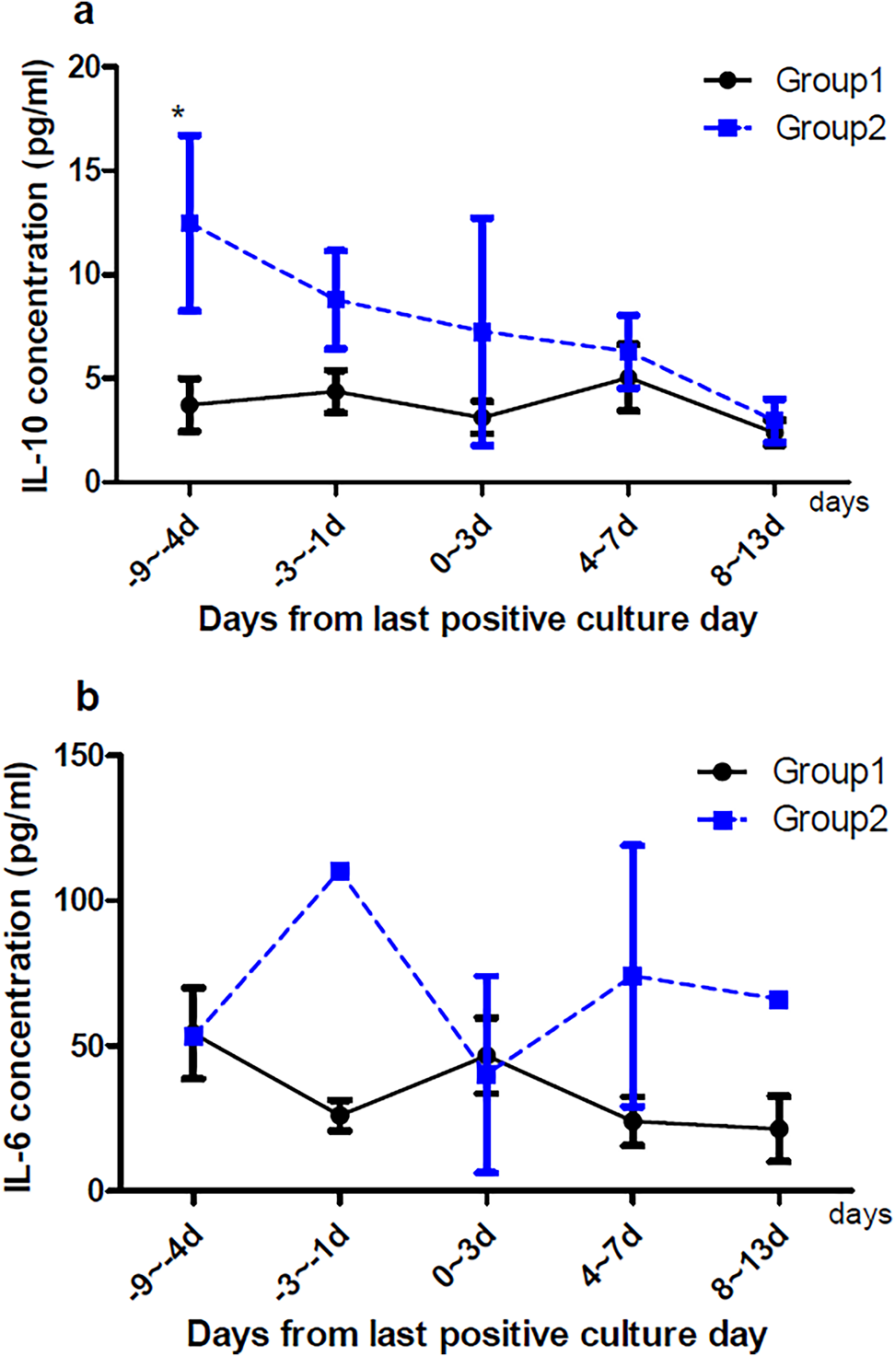
Comparison of cytokine concentrations in groups 1 and 2 in serial blood samples collected during the study period. (**A**) IL-10 concentration in plasma. Group 1 comprises 15 patients, and group 2 comprises four patients. (**B**) IL-6 concentration in plasma. Group 1 comprises 18 patients, and group 2 comprises 4 patients. Day 0 was the last day of positive culture. *p < 0.05. Group 1 comprises patients with a Pitt bacteremia score of < 4. Group 2 comprises patients with a Pitt bacteremia score of ≥ 4.

**Figure 3:**
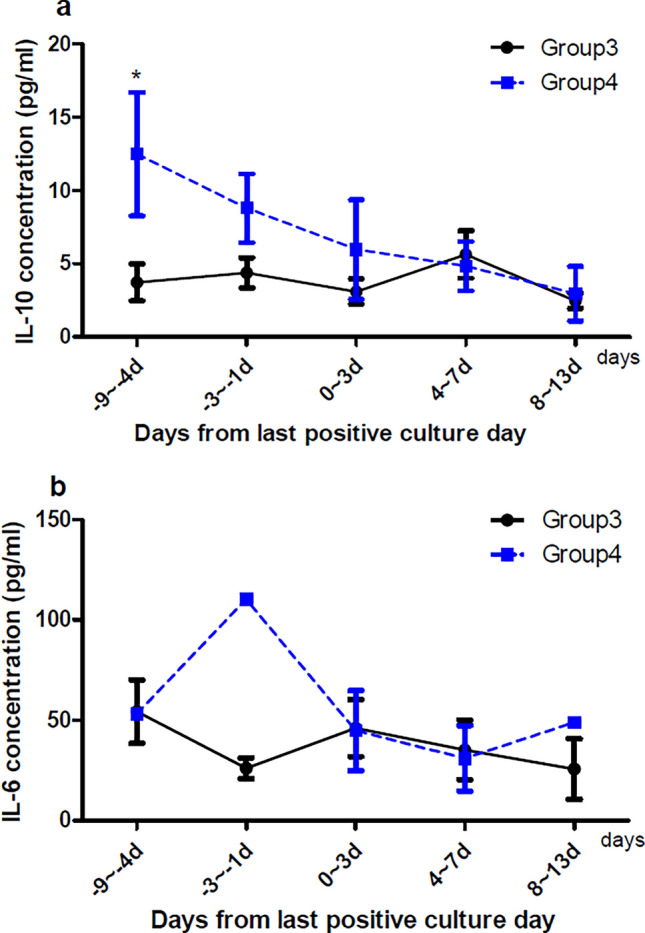
Comparison of cytokine concentrations in group 3 and 4 in serial blood samples collected during the study period. (**A**) IL-10 concentration in plasma. Group 3 comprises 15 patients, and group 4 comprises four patients. (**B**) IL-6 concentration in plasma. Group 3 comprises 18 patients, and group 4 comprises 4 patients. Day 0 was the last day of positive culture. *p < 0.05. Group 3 comprises patients who survived for 12 weeks from the index day. Group 4 comprises patients who died within 12 weeks from the index day.

We compared groups according to the Pitt bacteremia score and 12-week mortality (Figs. 2 and 3). IL-10 concentrations were higher in patients with high bacteremia scores than those with low bacteremia scores 4–9 days prior to the last positive culture day (Fig. 2, *p* < 0.05). The concentrations of IL-10 in the 4–9 days before the last positive culture day were higher in patients who died within 12 weeks from the index day than those who survived (Fig. 3, *p* < 0.05). IL-6 concentrations were generally higher in patients with higher Pitt bacteremia scores and those who died within 12 weeks, but the differences between groups did not reach statistical significance.

### Kinetics of IFN-γ and CD4^+^ T cells secreting IFN-γ in patients with PB

**Figure 4:**
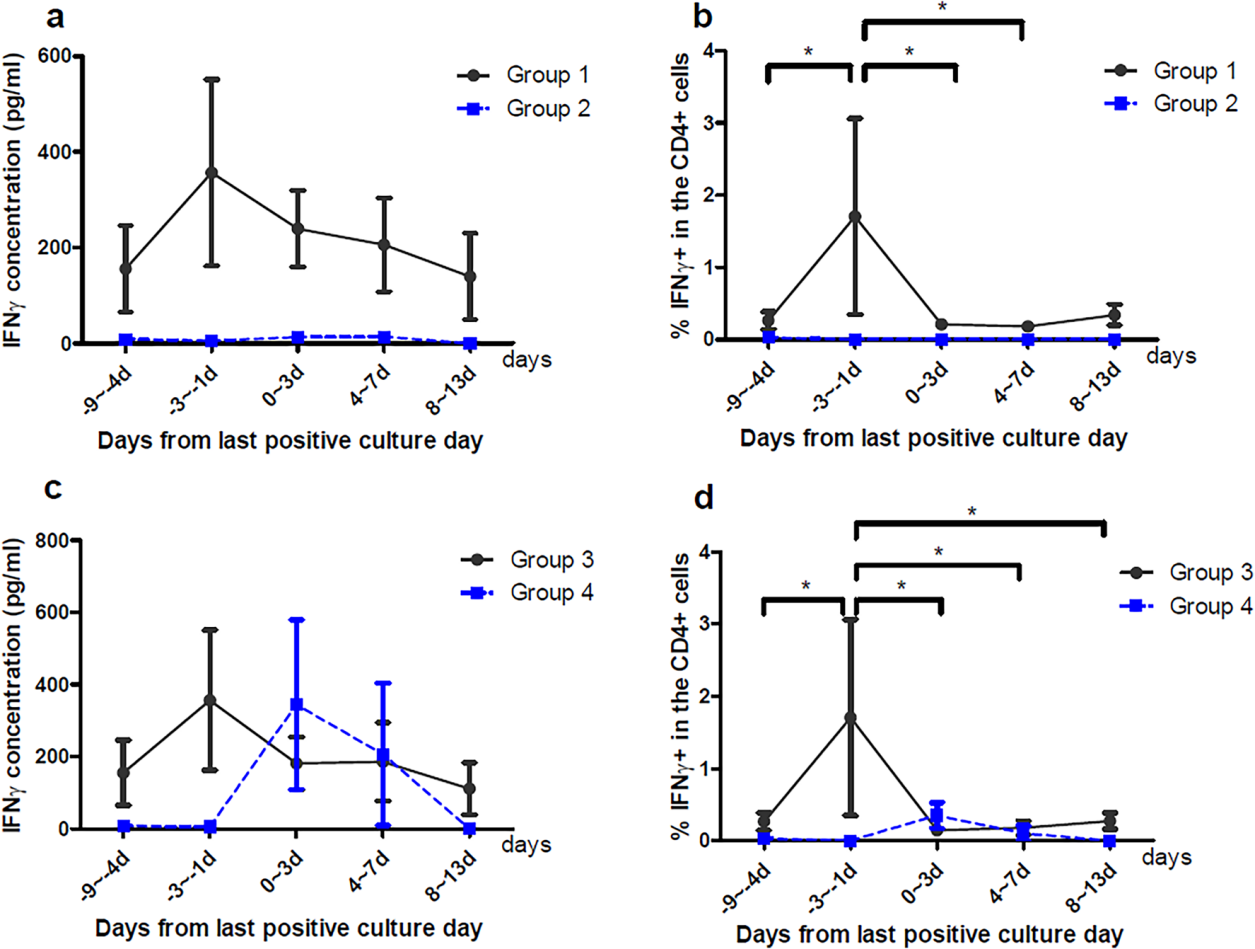
Comparison between groups of IFNγ concentrations and the proportion of IFNγ-secreting cells among the CD4^+^ T cells in patients with serial blood samples collected during the study period. PBMCs were collected from 22 patients and stimulated with heat-killed *S. aurerus*, and the concentration of IFNγ was measurable in 19 patients. In addition, the proportion of IFNγ-secreting cells among the CD4^+^ T cells was assessed using flow cytometry for 16 patients. (**A**) IFNγ concentration in Group 1 and Group 2. (**B**) The proportion of IFNγ-secreting cells among CD4^+^ T cells in Group 1 and Group 2. (**C**) IFNγ concentration in Group 3 and Group 4. (**D**) the proportion of IFNγ-secreting cells among CD4^+^ T cells in Group 3 and Group 4. Day 0 was the last day of positive culture. *p < 0.05. Group 1 comprises patients with Pitt bacteremia score < 4. Group 2 comprises patients with Pitt bacteremia ≥ 4. Group 3 comprises patients who survived for 12 weeks from the index day. Group 4 comprises patients who died within 12 weeks from the index day.

IFN-γ was undetectable in the plasma of 22 patients from whom blood samples were collected periodically (Supplementary Table S4). We collected PBMCs from these 22 patients to analyze IFN-γ further. After stimulation of the PBMCs with heat-killed *S. aureus*, the IFN-γ in the cell culture medium was detected for PBMCs isolated from 19 patients. IFN-γ concentrations increased until just before the blood cultures underwent a negative conversion and decreased after the last positive blood culture day for 15 patients with low bacteremia scores (Fig. 4a). IFN-γ concentrations were very low for all time points for four patients with high bacteremia scores.

**Figure 5:**
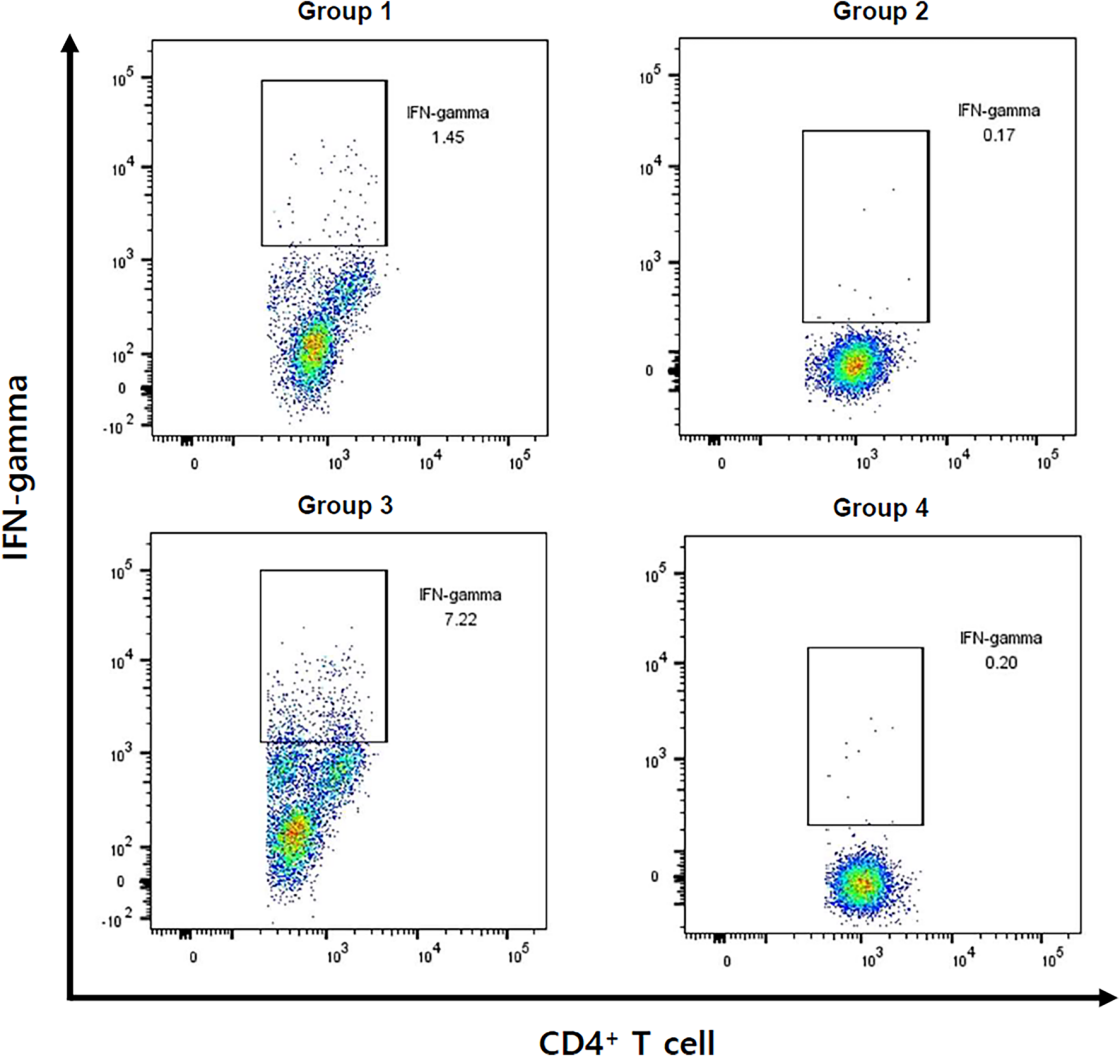
Proportion of IFNγ-secreting cells among CD4^+^ T cells analyzed using flow cytometry 1–3 days before the last positive culture day. Group 1 comprises 15 patients, and group 2 comprises one patient. Group 3 comprises 13 patients, and group 4 comprises 3 patients. Group 1 comprises patients with Pitt bacteremia score < 4. Group 2 comprises patients with Pitt bacteremia ≥ 4. Group 3 comprises patients who survived for 12 weeks from the index day. Group 4 comprises patients who died within 12 weeks from the index day.

To determine whether CD4^+^ T cells produce IFN-γ, intracellular staining was performed, and IFN-γ secreting cells among the CD4^+^ T cells of 16 patients could be analyzed by using flow cytometry. The proportion of IFN-γ secreting CD4^+^ T cells tended to be correlated with the IFN-γ concentration and was significantly high 1–3 days before the last positive culture day for 15 patients with low bacteremia scores (Fig. 4b, group 1). IFN-γ secreting CD4^+^ T cells were identified for only one patient with a high bacteremia score (Fig. 4b, group 2). When comparing groups of patients according to the 12-week mortality, the concentration of IFN-γ increased until the blood culture became negative and decreased after the last positive culture day in both groups (Fig. 4c). In addition, the proportion of IFN-γ secreting cells among the CD4^+^ T cells was the highest 1–3 days before the blood culture became negative for 13 patients who survived for 12 weeks from the index day (Fig. 4d, group 3). Figure 5 shows the proportion of IFN-γ secreting cells among the CD4^+^ T cells analyzed using flow cytometry 1–3 days before the last positive culture day.

## Discussion

In this study, we evaluated the risk factors associated with mortality in patients with SAB PB and confirmed a T cell immune response during *S. aureus* infection. In particular, our study is meaningful in that it sequentially observed changes in cytokines and T cell responses over time in human SAB. The APACHE II score and female sex were independent risk factors of 30 days mortality. The subgroup analysis according to MRSA isolates showed that the APACHE II score, liver cirrhosis, and a vancomycin MIC of ≥ 1.5 mg/L were independent risk factors for 30 days mortality. The IL-10 concentration was high in the early phase of bacteremia in patients with high Pitt bacteremia scores and those who died within 12 weeks from the index day. The proportion of IFN-γ-secreting CD4^+^ T cells was highest just before the blood cultures turned negative for patients with low Pitt bacteremia scores and those who survived for 12 weeks.

Previously, there have been several studies showing the risk factors of SAB PB. Patients with liver cirrhosis have impaired immunity and increased susceptibility to *S. aureus* infections. *S. aureus* is an important pathogen in liver cirrhosis patients^31,32^. Liver cirrhosis is a risk factor for PB compared with RB; moreover, liver cirrhosis patients with SAB had a higher mortality rate than non-liver cirrhosis patients in previous studies^8,33^. A high vancomycin MIC is also associated with worse clinical outcomes and treatment failure but there has been a controversy about whether a high vancomycin MIC is associated with persistent bacteremia^4,8,34,35^. Sheng-Hsiang Lin et al.^36^ showed that a decreased susceptibility to vancomycin was associated with mortality in MRSA PB. In this study, we evaluated the risk factors associated with 30 days mortality in patients with SAB PB. Liver cirrhosis was identified as a risk factor for 30 days mortality in patients with MRSA PB. In addition, the surviving patients were more likely to have a lower vancomycin MIC, and a vancomycin MIC of ≥ 1.5 mg/L was an independent risk factor for 30 days mortality from MRSA PB. Decreased susceptibility to vancomycin appears to affect the mortality of patients with MRSA PB treated with glycopeptides including vancomycin.

The *agr* locus of *S. aureus* globally controls the coordinated production of virulence factors^27^. Mutations in the *agr* locus cause *agr* dysfunction, alter the expression of autolysins and hemolysins, and have a global effect on bacterial pathogenicity^37,38^. *agr* dysfunction has been associated with PB, vancomycin bactericidal activity, and the mortality of SAB patients^6,28,39^. Loss of *agr* function appears to decrease the susceptibility to glycopeptides^40^. Moise-broder et al.^41^ reported that *agr* group II was a predictor of vancomycin treatment failure of MRSA bacteremia and Park et al.^42^ showed that *agr* group II was more pronounced in MRSA isolates with a vancomycin MIC of 2 mg/L. In the present study, *agr* dysfunction was noted in 64.9% of MRSA PB cases and there was no difference between the two groups. *agr* group II was the most common genotype of MRSA PB, and although statistically insignificant, *agr* group II was more frequent in patients who died within 30 days from the index day than in surviving patients. Additional studies are necessary to investigate further the association of mortality in PB patients with the *agr* group and *agr* dysfunction.

Pro-inflammatory cytokines such as IL-6, tissue necrosis factor (TNF), and IL-17A are necessary for initiating an effective inflammatory process against infection and they are associated with multiple-system organ failure and mortality^43^. IL-10 is an anti-inflammatory cytokine that regulates the immune response to pathogens^44^. Previous studies have tried to show a correlation between cytokines and SAB. McNicholas et al.^12^ reported that IL-6, a pro-inflammatory cytokine, might be an early inflammatory marker of complicated SAB. IL-10 was found to be associated with mortality and persistent bacteremia^10,11,13^. It is significant that, unlike previous studies, we measured IL-6 and IL-10 concentrations serially throughout the bacteremia period. Although there was no significant difference, the plasma concentration of IL-6 generally tended to be high in patients with high Pitt bacteremia scores and those who died within 12 weeks, before the blood culture became negative. The plasma concentration of IL-10 4–8 days before the last positive blood culture day was significantly higher in patients with high bacteremia scores and patients who died within 12 weeks from the index day. The IL-10 concentration in the early stage of bacteremia can be a predictive factor of poor clinical outcomes of persistent bacteremia.

There have been several lines of evidence showing that T cell immunity plays an important role in *S. aureus* infection. T lymphocyte-deficient mice were more susceptible to infection with *S. aureus*, and T cell-derived IFN-γ might be a pivotal regulator of neutrophil recruitment during *S. aureus* infection in a mouse model^45,46^. Human immunodeficiency virus infected patients with impaired Th1 cell immunity and low CD4^+^ counts are highly susceptible to *S. aureus* and have more invasive infections and a higher recurrence rate than patients with intact T cell immunity^47–51^. The first study showing the activation of a T cell immune response in human *S. aureus* infection was published in 2015. Brown et al*.*^30^ showed that human *S. aureus* blood stream infection induces *S. aureus* antigen-specific IFN- γ-producing CD4^+^ (Th1) cells.

In our study, we attempted to ascertain the T cell immune response within a real world clinical setting in patients with SAB PB. After stimulation of PBMCs from PB patients with heat-killed *S. aureus*, we were able to analyze the concentrations of secreted IFN-γ in the cell culture medium. The concentration of IFN-γ was highest 1–3 days before the blood culture became negative, and it decreased after the last positive blood culture day in patients with low disease severity compared to those with severe disease. In addition, the proportion of IFN-γ-producing CD4^+^ Th1 cells showed similar kinetics to the IFN-γ concentration during infection. Translating these findings, we found that CD4^+^ T cells are an important source of IFN-γ production during bacteremia and they play an important role in bacterial clearance and contribute to disease severity in SAB PB. While previous studies have shown a potential for T cell immunity to play a protective role against SAB infection, our study further clarifies the role of T cell immunity in *S. aureus* PB by showing the kinetics of CD4^+^ T cells during *S. aureus* bacteremia.

Our study has several limitations. First, the patients with a long duration of bacteremia were likely to be managed in great detail by infectious disease specialists, which included receiving additive diagnostic tests and antibiotic treatment. These actions might have resulted in a bias against the outcome of PB. Second, because the definition of PB is seven days, the possibility of an immortal time bias cannot be overlooked. Further analysis is needed to reduce immortal time bias and determine the presence of differences in outcomes depending on the duration of bacteremia. Third, only a small number of PB patients had serial blood samples collected. Additionally, the cytokines of some patients were unable to be analyzed. It might be necessary to check whether these cytokines did not exist or their concentration was too low to be detected. We might be able to use methods such as amplification in the near future. In the case of IFN-γ, we could not detect IFN-γ directly in plasma and could analyze IFN-γ only after stimulation of PBMC with heat-killed *S. aureus*. It is conceivable that memory Th1 cells that were previously exposed to *S. aureus* were induced to produce IFN-γ. Additionally, in our other experiments, IFN-γ was more detectable in plasma collected early in bacteremia. This means that IFN-γ levels in plasma can be affected by the bacterial concentration and antibiotics treatment. Additional studies with a larger sample size might be required to clarify the role of cytokines and T cell immunity during *S. aureus* bacteremia. Despite these limitations, this study is meaningful in that it is the first study to demonstrate the kinetics of T cell responses and describe the role of IFN-γ-producing CD4^+^ Th1 cells in a specific clinical setting of human *S. aureus* PB.

In summary, this study evaluated the risk factors for mortality of persistent SAB and the T cell immune response during SAB PB in a large cohort over 13 years. Clinical factors such as APACH II score in PB, liver cirrhosis, and vancomycin MIC in MRSA PB were associated with mortality. An elevated IL-10 concentration was predictive of mortality from PB. The most significant finding is that IFN-γ producing CD4^+^ T cells play an important role in bacterial clearance during bacteremia and affect disease severity. The current study will help understand the T cell immune response to *S. aureus* infection in humans and will guide further studies on SAB.

## Supplementary Information

Supplementary Tables.

## Data Availability

The data of this study are available from the corresponding author upon reasonable request.

## References

[1] Wyllie, D. H., Crook, D. W. & Peto, T. E. Mortality after *Staphylococcus aureus* bacteraemia in two hospitals in Oxfordshire, 1997–2003: Cohort study. *BMJ* **333**, 281. 10.1136/bmj.38834.421713.2F (2006). 16798756 10.1136/bmj.38834.421713.2F PMC1526943

[2] van Hal, S. J. *et al.* Predictors of mortality in *Staphylococcus aureus* bacteremia. *Clin. Microbiol. Rev.* **25**, 362–386. 10.1128/cmr.05022-11 (2012). 22491776 10.1128/CMR.05022-11 PMC3346297

[3] Khatib, R. *et al.* Persistent *Staphylococcus aureus* bacteremia: Incidence and outcome trends over time. *Scand. J. Infect. Dis.* **41**, 4–9. 10.1080/00365540802441711 (2009). 18821135 10.1080/00365540802441711

[4] Hawkins, C. *et al.* Persistent *Staphylococcus aureus* bacteremia: An analysis of risk factors and outcomes. *Arch. Intern. Med.* **167**, 1861–1867. 10.1001/archinte.167.17.1861 (2007). 17893307 10.1001/archinte.167.17.1861

[5] Khatib, R. *et al.* Persistence in *Staphylococcus aureus* bacteremia: Incidence, characteristics of patients and outcome. *Scand. J. Infect. Dis.* **38**, 7–14. 10.1080/00365540500372846 (2006). 16338832 10.1080/00365540500372846

[6] Fowler, V. G. Jr. *et al.* Persistent bacteremia due to methicillin-resistant *Staphylococcus aureus* infection is associated with agr dysfunction and low-level in vitro resistance to thrombin-induced platelet microbicidal protein. *J. Infect. Dis.* **190**, 1140–1149. 10.1086/423145 (2004). 15319865 10.1086/423145

[7] Chang, F. Y. *et al.* A prospective multicenter study of Staphylococcus aureus bacteremia: Incidence of endocarditis, risk factors for mortality, and clinical impact of methicillin resistance. *Medicine (Baltimore)* **82**, 322–332. 10.1097/01.md.0000091185.93122.40 (2003). 14530781 10.1097/01.md.0000091185.93122.40

[8] Chong, Y. P. *et al.* Persistent *Staphylococcus aureus* bacteremia: A prospective analysis of risk factors, outcomes, and microbiologic and genotypic characteristics of isolates. *Medicine (Baltimore)* **92**, 98–108. 10.1097/MD.0b013e318289ff1e (2013). 23429353 10.1097/MD.0b013e318289ff1e PMC4553980

[9] Minejima, E. *et al.* Defining the breakpoint duration of *Staphylococcus aureus* bacteremia predictive of poor outcomes. *Clin. Infect. Dis.* **70**, 566–573. 10.1093/cid/ciz257 (2020). 30949675 10.1093/cid/ciz257 PMC7768749

[10] Minejima, E. *et al.* A dysregulated balance of proinflammatory and anti-inflammatory host cytokine response early during therapy predicts persistence and mortality in *Staphylococcus aureus* bacteremia. *Crit. Care Med.* **44**, 671–679. 10.1097/ccm.0000000000001465 (2016). 26540400 10.1097/CCM.0000000000001465 PMC6504958

[11] Rose, W. E. *et al.* Increased endovascular *Staphylococcus aureus* inoculum is the link between elevated serum interleukin 10 concentrations and mortality in patients with bacteremia. *Clin. Infect. Dis.* **64**, 1406–1412. 10.1093/cid/cix157 (2017). 28205673 10.1093/cid/cix157 PMC5411397

[12] McNicholas, S. *et al.* Cytokine responses to *Staphylococcus aureusbloodstream* infection differ between patient cohorts that have different clinical courses of infection. *BMC Infect. Dis.* **14**, 580. 10.1186/s12879-014-0580-6 (2014). 25398383 10.1186/s12879-014-0580-6 PMC4237739

[13] Volk, C. F. *et al.* Interleukin (IL)-1β and IL-10 host responses in patients with *Staphylococcus aureus* bacteremia determined by antimicrobial therapy. *Clin. Infect. Dis.* **70**, 2634–2640. 10.1093/cid/ciz686 (2020). 31365924 10.1093/cid/ciz686 PMC7286365

[14] Bröker, B. M., Mrochen, D. & Péton, V. The T cell response to *Staphylococcus aureus*. *Pathogens*10.3390/pathogens5010031 (2016). 26999219 10.3390/pathogens5010031 PMC4810152

[15] Spellberg, B. & Daum, R. Development of a vaccine against *Staphylococcus aureus*. *Semin. Immunopathol.* **34**, 335–348. 10.1007/s00281-011-0293-5 (2012). 22080194 10.1007/s00281-011-0293-5 PMC4184131

[16] Miller, L. S., Fowler, V. G., Shukla, S. K., Rose, W. E. & Proctor, R. A. Development of a vaccine against *Staphylococcus aureus* invasive infections: Evidence based on human immunity, genetics and bacterial evasion mechanisms. *FEMS Microbiol. Rev.* **44**, 123–153. 10.1093/femsre/fuz030 (2020). 31841134 10.1093/femsre/fuz030 PMC7053580

[17] Zhao, Y. X., Nilsson, I. M. & Tarkowski, A. The dual role of interferon-gamma in experimental *Staphylococcus aureus* septicaemia versus arthritis. *Immunology* **93**, 80–85. 10.1046/j.1365-2567.1998.00407.x (1998). 9536122 10.1046/j.1365-2567.1998.00407.x PMC1364109

[18] McLoughlin, R. M., Lee, J. C., Kasper, D. L. & Tzianabos, A. O. IFN-gamma regulated chemokine production determines the outcome of *Staphylococcus aureus* infection. *J. Immunol.* **181**, 1323–1332. 10.4049/jimmunol.181.2.1323 (2008). 18606687 10.4049/jimmunol.181.2.1323

[19] Joshi, A. *et al.* Immunization with *Staphylococcus aureus* iron regulated surface determinant B (IsdB) confers protection via Th17/IL17 pathway in a murine sepsis model. *Hum. Vaccin. Immunother.* **8**, 336–346. 10.4161/hv.18946 (2012). 22327491 10.4161/hv.18946 PMC3426080

[20] Knaus, W. A., Draper, E. A., Wagner, D. P. & Zimmerman, J. E. APACHE II: A severity of disease classification system. *Crit. Care Med.* **13**, 818–829 (1985). 3928249

[21] Chow, J. W. *et al.* Enterobacter bacteremia: Clinical features and emergence of antibiotic resistance during therapy. *Ann. Intern. Med.* **115**, 585–590. 10.7326/0003-4819-115-8-585 (1991). 1892329 10.7326/0003-4819-115-8-585

[22] Charlson, M. E., Pompei, P., Ales, K. L. & MacKenzie, C. R. A new method of classifying prognostic comorbidity in longitudinal studies: Development and validation. *J. Chronic Dis.* **40**, 373–383. 10.1016/0021-9681(87)90171-8 (1987). 3558716 10.1016/0021-9681(87)90171-8

[23] Friedman, N. D. *et al.* Health care-associated bloodstream infections in adults: A reason to change the accepted definition of community-acquired infections. *Ann. Intern. Med.* **137**, 791–797. 10.7326/0003-4819-137-10-200211190-00007 (2002). 12435215 10.7326/0003-4819-137-10-200211190-00007

[24] Li, J. S. *et al.* Proposed modifications to the Duke criteria for the diagnosis of infective endocarditis. *Clin. Infect. Dis.* **30**, 633–638. 10.1086/313753 (2000). 10770721 10.1086/313753

[25] Singer, M. *et al.* The third international consensus definitions for sepsis and septic shock (Sepsis-3). *JAMA* **315**, 801–810. 10.1001/jama.2016.0287 (2016). 26903338 10.1001/jama.2016.0287 PMC4968574

[26] Oliveira, D. C. & de Lencastre, H. Multiplex PCR strategy for rapid identification of structural types and variants of the mec element in methicillin-resistant *Staphylococcus aureus*. *Antimicrob. Agents Chemother.* **46**, 2155–2161. 10.1128/aac.46.7.2155-2161.2002 (2002). 12069968 10.1128/AAC.46.7.2155-2161.2002 PMC127318

[27] Shopsin, B. *et al.* Prevalence of agr specificity groups among *Staphylococcus aureus* strains colonizing children and their guardians. *J. Clin. Microbiol.* **41**, 456–459. 10.1128/jcm.41.1.456-459.2003 (2003). 12517893 10.1128/JCM.41.1.456-459.2003 PMC149583

[28] Schweizer, M. L. *et al.* Increased mortality with accessory gene regulator (agr) dysfunction in *Staphylococcus aureus* among bacteremic patients. *Antimicrob. Agents Chemother.* **55**, 1082–1087. 10.1128/aac.00918-10 (2011). 21173172 10.1128/AAC.00918-10 PMC3067101

[29] Enright, M. C., Day, N. P., Davies, C. E., Peacock, S. J. & Spratt, B. G. Multilocus sequence typing for characterization of methicillin-resistant and methicillin-susceptible clones of *Staphylococcus aureus*. *J. Clin. Microbiol.* **38**, 1008–1015 (2000). 10698988 10.1128/jcm.38.3.1008-1015.2000 PMC86325

[30] Brown, A. F. *et al.* Memory Th1 cells are protective in invasive *Staphylococcus aureus* infection. *PLOS Pathogens* **11**, e1005226. 10.1371/journal.ppat.1005226 (2015). 26539822 10.1371/journal.ppat.1005226 PMC4634925

[31] Campillo, B., Richardet, J. P., Kheo, T. & Dupeyron, C. Nosocomial spontaneous bacterial peritonitis and bacteremia in cirrhotic patients: Impact of isolate type on prognosis and characteristics of infection. *Clin. Infect. Dis.* **35**, 1–10. 10.1086/340617 (2002). 12060868 10.1086/340617

[32] Dupeyron, C., Campillo, S. B., Mangeney, N., Richardet, J. P. & Leluan, G. Carriage of *Staphylococcus aureus* and of gram-negative bacilli resistant to third-generation cephalosporins in cirrhotic patients: A prospective assessment of hospital-acquired infections. *Infect. Control Hosp. Epidemiol.* **22**, 427–432. 10.1086/501929 (2001). 11583211 10.1086/501929

[33] Park, H. J. *et al.* Clinical significance of Staphylococcus aureus bacteremia in patients with liver cirrhosis. *Eur. J. Clin. Microbiol. Infect. Dis.* **31**, 3309–3316. 10.1007/s10096-012-1697-4 (2012). 22833245 10.1007/s10096-012-1697-4

[34] van Hal, S. J., Lodise, T. P. & Paterson, D. L. The clinical significance of vancomycin minimum inhibitory concentration in *Staphylococcus aureus* infections: A systematic review and meta-analysis. *Clin. Infect. Dis.* **54**, 755–771. 10.1093/cid/cir935 (2012). 22302374 10.1093/cid/cir935

[35] Soriano, A. *et al.* Influence of vancomycin minimum inhibitory concentration on the treatment of methicillin-resistant *Staphylococcus aureus* bacteremia. *Clin. Infect. Dis.* **46**, 193–200. 10.1086/524667 (2008). 18171250 10.1086/524667

[36] Lin, S. H. *et al.* Risk factors for mortality in patients with persistent methicillin-resistant *Staphylococcus aureus* bacteraemia in a tertiary care hospital in Taiwan. *J. Antimicrob. Chemother.* **65**, 1792–1798. 10.1093/jac/dkq188 (2010). 20511366 10.1093/jac/dkq188

[37] Fujimoto, D. F. & Bayles, K. W. Opposing roles of the Staphylococcus aureus virulence regulators, Agr and Sar, in Triton X-100- and penicillin-induced autolysis. *J. Bacteriol.* **180**, 3724–3726. 10.1128/jb.180.14.3724-3726.1998 (1998). 9658022 10.1128/jb.180.14.3724-3726.1998 PMC107347

[38] Villaruz, A. E. *et al.* A point mutation in the *agr* locus rather than expression of the panton-valentine leukocidin caused previously reported phenotypes in *Staphylococcus aureus* pneumonia and gene regulation. *J. Infect. Dis.* **200**, 724–734. 10.1086/604728 (2009). 19604047 10.1086/604728 PMC2777534

[39] Sakoulas, G. *et al.* Reduced susceptibility of *Staphylococcus aureus* to vancomycin and platelet microbicidal protein correlates with defective autolysis and loss of accessory gene regulator (*agr*) function. *Antimicrob. Agents Chemother.* **49**, 2687–2692. 10.1128/aac.49.7.2687-2692.2005 (2005). 15980337 10.1128/AAC.49.7.2687-2692.2005 PMC1168700

[40] Sakoulas, G. *et al.* Accessory gene regulator (*agr*) locus in geographically diverse *Staphylococcus aureus* isolates with reduced susceptibility to vancomycin. *Antimicrob. Agents Chemother.* **46**, 1492–1502. 10.1128/aac.46.5.1492-1502.2002 (2002). 11959587 10.1128/AAC.46.5.1492-1502.2002 PMC127153

[41] Moise-Broder, P. A. *et al.* Accessory gene regulator group II polymorphism in methicillin-resistant *Staphylococcus aureus* is predictive of failure of vancomycin therapy. *Clin. Infect. Dis.* **38**, 1700–1705. 10.1086/421092 (2004). 15227615 10.1086/421092

[42] Park, M.-J. *et al.* Accessory gene regulator polymorphism and vancomycin minimum inhibitory concentration in methicillin-resistant *Staphylococcus aureus*. *Ann. Lab. Med.* **35**, 399–403. 10.3343/alm.2015.35.4.399 (2015). 26131410 10.3343/alm.2015.35.4.399 PMC4446577

[43] Pinsky, M. R. *et al.* Serum cytokine levels in human septic shock. Relation to multiple-system organ failure and mortality. *Chest* **103**, 565–575. 10.1378/chest.103.2.565 (1993). 8432155 10.1378/chest.103.2.565

[44] Zhang, J.-M. & An, J. Cytokines, inflammation, and pain. *Int. Anesthesiol. Clin.* **45**, 27–37. 10.1097/AIA.0b013e318034194e (2007). 17426506 10.1097/AIA.0b013e318034194e PMC2785020

[45] Spellberg, B. *et al.* The antifungal vaccine derived from the recombinant N terminus of Als3p protects mice against the bacterium *Staphylococcus aureus*. *Infect. Immun.* **76**, 4574–4580. 10.1128/IAI.00700-08 (2008). 18644876 10.1128/IAI.00700-08 PMC2546811

[46] McLoughlin, R. M., Lee, J. C., Kasper, D. L. & Tzianabos, A. O. IFN-γ regulated chemokine production determines the outcome of *Staphylococcus aureus* infection. *J. Immunol.* **181**, 1323–1332. 10.4049/jimmunol.181.2.1323 (2008). 18606687 10.4049/jimmunol.181.2.1323

[47] Manfredi, R., Costigliola, P., Ricchi, E. & Chiodo, F. Sepsis-bacteraemia and other infections due to non-opportunistic bacterial pathogens in a consecutive series of 788 patients hospitalized for HIV infection. *Clin. Ter.* **143**, 279–290 (1993). 8258261

[48] Manfredi, R., Calza, L. & Chiodo, F. Epidemiology and microbiology of cellulitis and bacterial soft tissue infection during HIV disease: A 10-year survey. *J. Cutan. Pathol.* **29**, 168–172. 10.1034/j.1600-0560.2002.290307.x (2002). 11972714 10.1034/j.1600-0560.2002.290307.x

[49] Crum-Cianflone, N. F. *et al.* Trends and causes of hospitalizations among HIV-infected persons during the late HAART era: What is the impact of CD4 counts and HAART use?. *J. Acquir. Immune Defic. Syndr.* **54**, 248–257. 10.1097/qai.0b013e3181c8ef22 (2010). 20658748 10.1097/qai.0b013e3181c8ef22 PMC2911973

[50] Tong, S. Y. C., Davis, J. S., Eichenberger, E., Holland, T. L. & Fowler, V. G. Staphylococcus aureus Infections: Epidemiology, pathophysiology, clinical manifestations, and management. *Clin. Microbiol. Rev.* **28**, 603–661. 10.1128/CMR.00134-14 (2015). 26016486 10.1128/CMR.00134-14 PMC4451395

[51] Graber, C. J., Jacobson, M. A., Perdreau-Remington, F., Chambers, H. F. & Diep, B. A. Recurrence of skin and soft tissue infection caused by methicillin-resistant *Staphylococcus aureus* in a HIV primary care clinic. *JAIDS J. Acquir. Immune Defic. Syndr.* **49**, 231–233. 10.1097/QAI.0b013e318183a947 (2008). 18820536 10.1097/QAI.0b013e318183a947

