# Author Correction: Clinical and microbiological characteristics of persistent *Staphylococcus aureus* bacteremia, risk factors for mortality, and the role of CD4^+^ T cells

**DOI:** 10.1038/s41598-025-95193-6

**Published:** 2025-04-08

**Authors:** Eunmi Yang, Yeong Geon Cho, Eunsil Kim, Euijin Chang, Seongman Bae, Jiwon Jung, Min Jae Kim, Yong Pil Chong, Sung-Han Kim, Sang-Ho Choi, Sang-Oh Lee, Yun Shin Chung, Yang Soo Kim

**Affiliations:** 1 Division of Infectious Diseases, Seoul Medical Center, Seoul, South Korea; 2 Center for Antimicrobial Resistance and Microbial Genetics, University of Ulsan College of Medicine, Seoul, South Korea; 3 Asan Institute for Life Science, Asan Medical Center, Seoul, South Korea; 4 Division of Infectious Diseases, Asan Medical Center, University of Ulsan College of Medicine, 88, Olympic-ro43-gil, Songpa-gu, Seoul, 05505 South Korea

Correction to: *Scientific Reports* 10.1038/s41598-024-66520-0, published online 05 July 2024

The Funding section in the original version of this Article was incomplete.

“This work was supported by a grant from the Korea Health Technology R&D Project through the Korea Health Industry Development Institute (KHIDI) funded by the Ministry of Health & Welfare, Republic of Korea [Grant Number HV22C2029].”

now reads:

“This work was supported by a grant from the Korea Health Technology R&D Project through the Korea Health Industry Development Institute (KHIDI) funded by the Ministry of Health & Welfare, Republic of Korea [Grant Number HV22C0209].”

The original Article has been corrected.

