# Supplementary material for: Clinical and microbiological characteristics of persistent Staphylococcus aureus bacteremia, risk factors for mortality, and the role of CD4+ T cells
Source: Sci Rep. 2024 Jul 5;14:15472. doi: 10.1038/s41598-024-66520-0 (PMC11226624; doi:10.1038/s41598-024-66520-0)
Supplement: Supplementary file 1 — Supplementary Tables. [file 41598_2024_66520_MOESM1_ESM.doc]

**Supplementary Table S1. Vancomycin MIC and Clinical Management of Patients with Persistent MRSA Bacteremia**

| **Characteristic** | **Deceased patients**  **(n = 36) No. (%)** | **Surviving patients**  **(n = 149) No. (%)** | | ***p* value** |
| --- | --- | --- | --- | --- |
| **Vancomycin MIC by BMD** |  | |  |  |
| <1.5 mg/L | 20 (55.6) | | 112 (75.2) | 0.02 |
| ≥1.5 mg/L | 16 (44.4) | | 37 (24.8) | 0.02 |
| **Appropriate empirical antibiotic treatment**† | 29 (80.6) | | 103 (69.1) | 0.17 |
| Time to initiation of appropriate antibiotics (d), median (IQR) | 0 (0–1) | | 1 (0–2) | 0.04 |

MIC, minimum inhibitory concentration; MRSA, methicillin-resistant *Staphylococcus aureus*; BMD, broth microdilution method

† Administration of at least one effective antibiotic against the organism within 24 h after the index blood culture was considered appropriate empirical treatment. The antibiotics used were vancomycin, teicoplanin, and linezolid.

**Supplementary Table S2. Genotypes and *agr* Dysfunction Distribution in MRSA Isolates Responsible for Persistent Bacteremia**

| **Characteristic** | | **Deceased patients**  **(n = 36) No. (%)** | **Surviving patients**  **(n = 149) No. (%)** | ***p* value** |
| --- | --- | --- | --- | --- |
| **MLST CC** | **Sequence type** |  |  |  |
| CC5 | ST5 | 26 (72.2) | 83 (55.7) | 0.07 |
| CC8 | ST72 | 8 (22.2) | 45 (30.2) | 0.34 |
|  | ST239 | 1 (2.8) | 8 (5.4) | > 0.99 |
| others | | 1 (2.8) | 13 (8.7) | NA |
| **SCC*mec* type** | |  |  |  |
| II | | 26 (72.2) | 87 (58.4) | 0.13 |
| III | | 1 (2.8) | 10 (6.7) | 0.69 |
| IV | | 9 (25.0) | 50 (33.6) | 0.32 |
| NID | | 0 | 2 (1.3) | > 0.99 |
| ***agr* genotype** | |  |  |  |
| I | | 10 (27.8) | 60 (40.3) | 0.17 |
| II | | 26 (72.2) | 82 (55.0) | 0.06 |
| III | | 0 | 3 (2.0) | > 0.99 |
| IV | | 0 | 1 (0.7) | > 0.99 |
| NID | | 0 | 3 (2.0) | > 0.99 |
| ***agr* dysfunction** | | 26 (72.2) | 94 (63.1) | 0.30 |
| **ST5-SCC*mec* II** | | 26 (72.2) | 83 (55.7) | 0.07 |
| **ST72-SCC*mec*** **IV** | | 8 (22.2) | 45 (30.2) | 0.34 |

MLST, multi-locus sequence type; CC, clonal complex; ST, sequence type; SCC, staphylococcal cassette chromosome; NID, not identified

**Supplementary Table S3. Multivariate Analysis of Risk Factors for 30-day Mortality in Patients with** Persistent MRSA Bacteremia

|  | **Univariate analysis** | | | **Multivariate analysis**† | |
| --- | --- | --- | --- | --- | --- |
| **Characteristic** | **Deceased patients**  **(n = 36)** | **Surviving patients**  **(n = 149)** | ***p* value** | **Odds ratio**  **(95% CI)** | ***p* value** |
| Age | 60.5 (52.3–73.8) | 66 (58–72) | 0.19 |  |  |
| APACHE II, median (IQR) | 19.5 (15–26) | 7 (12–21) | 0.03 | 1.07 (1.01–1.13) | 0.03 |
| Community-acquired infection | 1 (2.8) | 19 (12.8) | 0.13 |  |  |
| Diabetes mellitus | 9 (25.0) | 54 (36.2) | 0.20 |  |  |
| Liver cirrhosis | 10 (27.8) | 17 (11.4) | 0.01 | 3.77 (1.43–9.95) | 0.01 |
| Male | 20 (55.6) | 101 (67.8) | 0.17 |  |  |
| Metastatic bone and joint infection | 0 | 22 (14.8) | 0.01 |  |  |
| Primary bacteremia | 4 (11.1) | 4 (2.7) | 0.048 |  |  |
| Vancomycin MIC ≥1.5 mg/L | 16 (44.4) | 37 (24.8) | 0.01 | 3.17 (1.39–7.25) | 0.01 |
| Time to initiation of appropriate antibiotics | 0 (0–1) | 1 (0–2) | 0.04 |  |  |

† This model fits the data well in terms of discrimination (C-statistic 0.741) and calibration (Hosmer-Lemeshow goodness-of-fit statistic 4.853; p = 0.773).

**Supplementary Table S4. Information on clinical background and outcomes of patients from whom serial blood samples were taken**

| Patients | Age | Sex | Underlying Disease | Pathogen | Site of infection | Treatment | SAB duration | Pitt score | Group† | 12-week outcome |
| --- | --- | --- | --- | --- | --- | --- | --- | --- | --- | --- |
| Patient 1 | 75 | M | DM, HTN | MRSA | Vascular graft infection -> focus remove | Vancomycin | 17 days | 0 | G1, G3 | Alive |
| Patient 2 | 58 | M | HTN, ESRD | MRSA | Vascular graft infection -> focus remove | Vancomycin -> linezolid | 19 days | 3 | G1, G3 | Alive |
| Patient 3 | 59 | M | DM | MRSA | Bone and joint infection | Vancomycin | 12 days | 0 | G1, G3 | Alive |
| Patient 4 | 38 | M | Atopic dermatitis | MRSA | Skin and soft tissue infection | Vancomycin | 7 days | 0 | G1, G3 | Alive |
| Patient 5 | 61 | M | DM, HTN, HCC | MSSA | Primary bacteremia | Nafcillin | 22 days | 0 | G1, G3 | Alive |
| Patient 6 | 66 | F | Breast cancer | MSSA | Bone and joint infection | Nafcillin | 10 days | 0 | G1, G3 | Alive |
| Patient 7 | 58 | M | None | MSSA | Urinary track infection | Nafcillin | 12 days | 1 | G1, G3 | Alive |
| Patient 8 | 65 | F | CVA, MS | MSSA | Surgical site infection | Nafcillin | 9 days | 1 | G1, G3 | Alive |
| Patient 9 | 64 | F | LC | MSSA | Infective endocarditis | Nafcillin | 15 days | 4 | G2, G4 | Dead |
| Patient 10 | 69 | M | CVA, HTN | MRSA | Catheter related infection -> focus remove | Teicoplanin | 13 days | 4 | G2, G4 | Dead |
| Patient 11 | 69 | M | Lung cancer, HTN | MRSA | Skin and soft tissue infection | Vancomycin -> linezolid | 74 days | 2 | G1, G4 | Dead |
| Patient 12 | 48 | M | DM | MSSA | Skin and soft tissue infection | Nafcillin | 11 days | 5 | G2, G3 | Alive |
| Patient 13 | 62 | M | LC, ESRD | MRSA | Bone and joint infection | Vancomycin | 8 days | 3 | G1, G4 | Dead |
| Patient 14 | 74 | M | ET | MRSA | Bone and joint infection | Linezolid | 9 days | 0 | G1, G3 | Alive |
| Patient 15 | 74 | M | HTN, COPD | MRSA | Aortic arch graft infection -> focus remove | Vancomycin | 22 days | 3 | G1, G3 | Alive |
| Patient 16 | 47 | M | CVA | MSSA | Infective endocarditis | Nafcillin | 8 days | 2 | G1, G3 | Alive |
| Patient 17 | 60 | M | DM | MSSA | Bone and joint infection -> focus remove | Nafcillin | 8 days | 0 | G1, G3 | Alive |
| Patient 18 | 71 | M | HTN | MRSA | Infective endocarditis -> focus remove | Vancomycin | 14 days | 2 | G1, G3 | Alive |
| Patient 19 | 37 | F | None | MRSA | Bone and joint infection | Vancomycin -> linezolid | 7 days | 2 | G1, G3 | Alive |
| Patient 20 | 57 | M | DM, HTN, LC | MRSA | Intra-abdominal infection | Vancomycin -> linezolid | 16 days | 2 | G1, G3 | Alive |
| Patient 21 | 70 | M | DM, HTN, HCC | MSSA | Primary bacteremia | Nafcillin | 7 days | 4 | G2, G3 | Alive |
| Patient 22 | 66 | F | Lung cancer, HTN | MSSA | Phlebitis | Nafcillin | 20 days | 0 | G1, G3 | Alive |

M, male; F, female; DM, diabetes mellitus; HTN, hypertension; MRSA, methicillin-resistant *Staphylococcus aureus*; ESRD, end stage renal disease; HCC, hepatocellular carcinoma; MSSA, methicillin-susceptible *Staphylococcus aureus*; CVA, cerebrovascular accident; MS, mitral valve stenosis; LC, liver cirrhosis; ET, essential thrombocythemia.

†Group: G1 comprises patients with Pitt bacteremia score < 4, G2 comprises patients with Pitt bacteremia ≥ 4, G3 comprises patients who survived for 12 weeks from the index day, and G4 comprises patients who died within 12 weeks from the index day.
